# Molecular evidence of rodent-associated mites tests the monophyly and challenges the cosmopolitan status of *Laelaps* Koch, 1836 (Mesostigmata: Laelapidae)

**DOI:** 10.1017/S0031182026102224

**Published:** 2026-06

**Authors:** Mario Espinoza-Carniglia, Ekaterina Savchenko, Ulyses F. J. Pardiñas, Marcela Lareschi

**Affiliations:** 1 Centro de Estudios Parasitológicos y de Vectores CEPAVE (CONICET-UNLP), La Plata, Argentina; 2 Instituto de Diversidad y Evolución Austral (IDEAus-CONICET), Puerto Madryn, Argentina; 3 Instituto Nacional de Biodiversidad (INABIO), Quito, Ecuador

**Keywords:** Acari, co-evolution, cosmopolitanism, Cricetidae, ectoparasites, Gamasida, host specificity, Muridae, polyphyly

## Abstract

The taxonomy of wide distributed parasites across continents has rarely been evaluated using molecular data, and it remains unclear whether their apparent broad distributions reflect true cosmopolitan and monophyletic lineages. There is also the expectation that long-term parasite–host associations shape the evolution of parasitic lineages and should therefore be reflected in parasite taxonomy. *Laelaps* Koch, 1836 (Mesostigmata: Laelapidae) is an example of a widespread genus, reported from multiple continents, parasitizing Cricetidae and Muridae rodents. The aim of this study is to evaluate whether *Laelaps* is monophyletic and truly broadly distributed across continents. To this end, we generated new nuclear (18S-ITS1-5.8S-ITS2-28S) and mitochondrial (cytochrome oxidase I) sequences from 8 species of *Laelaps*, and 4 related laelapid mite species of the genera *Androlaelaps* Berlese, 1903, *Gigantolaelaps* Fonseca, 1939 and *Mysolaelaps* Fonseca, 1936 associated with cricetid rodents from Argentina. Those sequences were analysed together with sequences from laelapids parasitizing native South American cricetids and from South Africa available in GenBank. Phylogenetic analyses revealed that *Laelaps* is polyphyletic. Within Cricetidae, tribal host association appears to drive phylogenetic structure, with cricetid-associated *Laelaps* lineages recovered as more closely related to *Gigantolaelaps* and *Mysolaelaps* than to each other. Murid-associated *Laelaps* from South Africa were recovered as lineages separated from cricetid-associated *Laelaps*, suggesting that the deep divergence between Cricetidae and Muridae may also shape laelapid phylogeny, a pattern that requires broader sampling to be conclusively evaluated. These results challenge the monophyly and broad distributional status of *Laelaps* and highlight the need for integrative taxonomic revision of this genus.

## Graphical abstract

**Figure:**
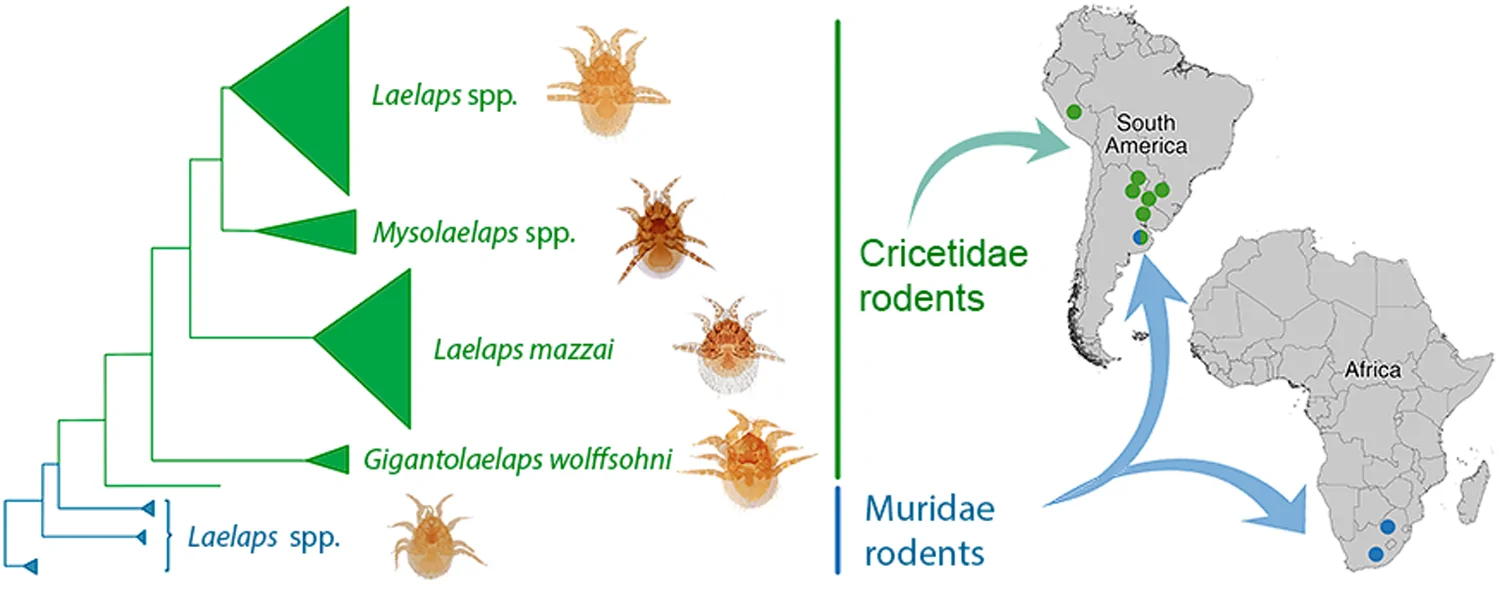

## Introduction

The classification of parasitic mites has historically relied heavily on conserved morphological characters, leading to the recognition of genera with remarkably broad geographic distributions and host ranges. Within Mesostigmata, this pattern is exemplified by genera such as *Androlaelaps* Berlese, 1903, *Laelaps* Koch, 1836 (Laelapidae) and *Ornithonyssus* Sambon, 1928 (Macronyssidae). These mite genera include species traditionally considered globally distributed and associated with a wide array of hosts (Tipton, 1960; Radovsky, 1985, 1994).

This study focuses on *Laelaps*, a genus reported from all continents, except Antarctica, parasitizing primarily rodents of the families Cricetidae and Muridae (Tipton, 1960; Domrow, 1964; Matthee et al., 2018; Savchenko and Lareschi, 2022; Yang et al., 2022; Lareschi, 2023). The genus was erected by Koch (1836) describing 2 species, *Laelaps agilis* and *Laelaps hilaris*, collected from murid and cricetid rodents, respectively, from Germany. Subsequently, *Laelaps* underwent multiple taxonomic revisions based on morphology that progressively refined the boundaries of the genus (Zachvatkin, 1948; Zumpt, 1950; Zumpt and Patterson, 1951; Bregetova, 1956; Evans and Till, 1966). However, these boundaries are still not entirely clear. Tipton (1960) and Furman and Tipton (1961) restricted *Laelaps sensu stricto*, excluding related laelapid genera (e.g. *Echinolaelaps* Ewing, 1929), whereas other authors treated *Echinolaelaps* and *Laelaps sensu stricto* as subgenera within a broader *Laelaps sensu lato* by incorporating additional morphological traits (see Strandtmann and Mitchell, 1963; Allred, 1969; Furman, 1971). This taxonomic framework has a certain degree of subjectivity, and it may have contributed to the long-standing perception of *Laelaps* as a single, widely distributed genus.

The reports of *Laelaps* in South America began with the descriptions of species, such as *Laelaps differens, Laelaps manguinhosi* and *Laelaps paulistanensis*, all associated with cricetid rodents from Brazil (Fonseca, 1936a). Currently, there is an uncertain number of *Laelaps* species recognized worldwide. Some species parasitize Old World murid rodents, whereas other are associated with South American cricetids (Fonseca, 1936a, 1939; Tipton, 1960; Furman, 1972a, b; Gettinger, 1992; Savchenko and Lareschi, 2022). This pattern has generally been interpreted as evidence of a single, broadly defined genus with a global distribution. However, this interpretation becomes problematic when viewed in an evolutionary context. Cricetidae and Muridae are sister families – if fossil taxa are excluded – that diverged around 25 million years ago and have since followed markedly different evolutionary trajectories, biogeographic histories and ecological associations (e.g. Steppan and Schenk, 2017; López-antoñanzas et al., 2024). Some species of murids, such as *Rattus rattus* (Linnaeus, 1758) and *Rattus norvegicus* (Berkenhout, 1769), have achieved global distributions largely through human-mediated dispersal and are strongly associated with anthropized environments (Steppan et al., 2005; Aghová et al., 2018; Byers et al., 2019). In contrast, cricetids underwent a rapid radiation in South America, remaining predominantly linked to natural or less anthropized habitats (Patton et al., 2015; Pardiñas et al., 2017; Ronez et al., 2023).

From an evolutionary perspective, the deep phylogenetic divergence of Cricetidae and Muridae raises doubts about the plausibility of long-term persistence of the same parasite mite lineages across both rodent families. Host–parasite co-evolutionary theory predicts that parasites with relatively strong host associations may tend to track host phylogeny over long evolutionary timescales, making true cosmopolitanism across deeply divergent host lineages unlikely (Hugot, 2007). Under this framework, the apparent cosmopolitan distribution of *Laelaps* may reflect historical reliance on morphology-based taxonomy rather than genuine evolutionary continuity.

Despite extensive documentation of host associations in *Laelaps*, phylogenetic relationships within the genus remain poorly explored, a gap particularly relevant given the role of its hosts as potential vectors of zoonotic pathogens (Bowen et al., 1997; Babolin et al., 2016). Previous molecular studies have suggested that host association may be influential in shaping *Laelaps* diversification. For example, host-specialist species such as *Laelaps giganteus* Berlese, 1918, show phylogenetic structure closely linked to murid hosts, whereas more generalist species such as *Laelaps muricola* Trägårdh, 1910 exhibit weaker host-associated patterns (Engelbrecht et al., 2014, 2016; Matthee et al., 2018). Similarly, Savchenko and Lareschi (2022) reported a dichotomy between *Laelaps* associated with South American cricetids and those parasitizing South African murids, although their analysis was based on a single molecular marker (18S-ITS1-5.8S-ITS2), leaving the evaluation of the monophyly of the genus unresolved.

The primary aim of this study is to evaluate the monophyly of the genus *Laelaps*. To this end, we integrate new molecular data from *Laelaps echidninus* Berlese, 1887 associated with *Rattus norvegicus* (Muridae) in Argentina, a recently introduced species in contact with native cricetids, together with sequences from laelapid mites parasitizing native cricetids from nearby localities and congeners from South Africa available in GenBank. This study design allows us to assess whether *Laelaps* is monophyletic and whether host association structures laelapid phylogeny.

## Materials and methods

### Studied material

Mites analysed in this study were collected by the authors and collaborators as part of broader research on rodents and parasites conducted in various localities across Argentina in previous years. Specimens were preserved in 96% ethanol and stored frozen at −20 °C. A total of 23 mites from 11 localities from Argentina (see Figure S1 in supplementary material) were selected from 15 rodents specimens representing the following species: Cricetidae: *Calomys callosus* (Rengger, 1830), *Calomys sorella* (Thomas, 1900), *Holochilus brasiliensis* (Desmarest, 1819), *Holochilus chacarius* Thomas, 1906, *Nectomys squamipes* (Brants, 1827), *Oligoryzomys nigripes* (von Olfers, 1818), *Oligoryzomys flavescens* (Waterhouse, 1837), *Oligoryzomys fornesi* (Massoia, 1973), *Scapteromys aquaticus* Thomas, 1920; Muridae: *Rattus norvegicus* (Berkenhout, 1769) (see details in Table 1).

**Table 1.** DNA sequences included in phylogenetic analysis of nuclear region 18S-ITS1-5.8S-ITS2-28S and cytochrome oxidase I (COI) Table 1 long description.

Mite species	Host species	Host family (tribe)	Country of origin of mites	18S-ITS1-5.8S-ITS2-28S	COI	Reference
*Dermanyssus gallinae*	*Gallus gallus*	Outgroup	Italy	EU375311	nd	Potenza et al., 2009
			Israel	nd	MH983858	Young et al., 2019
*Laelaps echidninus*	*Rattus norvegicus*	Muridae (Rattini)	Argentina	OQ241929*, OQ241930*	PV952220*, PV952221*	This study*
*Laelaps giganteus*	*Rhabdomys bechuane*	Muridae (Arvicanthini)	South Africa	MF419265, MF419267	KU166668, KU166669	Engelbrecht et al., 2014, 2016,
*Laelaps muricola*	*Mastomys coucha*	Muridae (Praomyini)	South Africa	MF419347, MF419348	KU166786, KU166787	Engelbrecht et al., 2014, 2016
*Laelaps scapteromyos*	*Scapteromys aquaticus*	Cricetidae (Akodontini)	Argentina	OK562186, OK562187, OK562188	PV952209*, PV952210*, PV952211*	Savchenko and Lareschi, 2022; this study*
*Laelaps galliarii*	*Oligoryzomys nigripes*	Cricetidae (Oryzomyini)	Argentina	OQ241939*, OQ241940*	OR405892*, OR405893*	This study*
*Laelaps manguinhosi*	*Holochilus brasiliensis*	Cricetidae (Oryzomyini)	Argentina	OK562180, OK562181	PV952213*, PV952214*	Savchenko and Lareschi, 2022; this study*
	*Holochilus chacarius*	Cricetidae (Oryzomyini)	Argentina	OQ241937*	PV952212*	
*Laelaps odysseos*	*Nectomys squamipes*	Cricetidae (Oryzomyini)	Argentina	OK562178	PV952208*	Savchenko and Lareschi, 2022; this study*
*Laelaps paulistanensis*	*Oligoryzomys nigripes*	Cricetidae (Oryzomyini)	Argentina	OQ241942*, OQ241943*	OR405894*, OR405895*	This study*
*Laelaps schatzi*	*Oligoryzomys flavescens*	Cricetidae (Oryzomyini)	Argentina	MK725870	MK716210, MK716211	Savchenko and Lareschi, 2019, 2022; this study*
	*Oligoryzomys fornesi*	Cricetidae (Oryzomyini)	Argentina	OK562194, OQ241941*	OR405898*	
*Laelaps mazzai*	*Calomys callidus*	Cricetidae (Phyllotini)	Argentina	OL514177, OL514178, OL514179	OL514187, OL514190, OL514191	Espinoza-Carniglia et al., 2023
	*Calomys callosus*	Cricetidae (Phyllotini)	Argentina	OL514174, OL514175, OL514176	OL514186, OL514188, OL514192	
	*Calomys fecundus*	Cricetidae (Phyllotini)	Argentina	OL514172	OL514185	
	*Calomys laucha*	Cricetidae (Phyllotini)	Argentina	OL514173	OL514189	
	*Calomys venustus*	Cricetidae (Phyllotini)	Argentina	ON847359, ON847360, ON847361	ON847362, ON847363, ON847364	
	*Calomys sorella*	Cricetidae (Phyllotini)	Peru	PQ276488	PV952215*	Espinoza-Carniglia et al., 2025; this study*
*Androlaelaps fahrenholzi*	*Calomys callosus*	Cricetidae (Phyllotini)	Argentina	PP693101	PV952216*	Savchenko et al., 2025; this study*
*Gigantolaelaps wolffsohni*	*Oligoryzomys flavescens*	Cricetidae (Oryzomyini)	Argentina	OK562177	PV852219*	Savchenko and Lareschi, 2022; this study*
	*Oligoryzomys nigripes*	Cricetidae (Oryzomyini)	Argentina	OQ241931, OQ241932	PV952217*, PV952218*	Savchenko et al., 2025; this study*
*Mysolaelaps microspinosus*	*Oligoryzomys flavescens*	Cricetidae (Oryzomyini)	Argentina	OQ241933, OQ241934	PV952202*, PV952205*	
*Mysolaelaps parvispinosus*	*Oligoryzomys flavescens*	Cricetidae (Oryzomyini)	Argentina	OQ241935, OQ241936	PV952206*, PV952207*	

Sequences obtained in this study are indicated with asterisks (*), no data (nd).

Following molecular procedures, mite exoskeletons were recovered from Chelex®100 solution, were cleared in lactophenol and mounted in Hoyer’s medium for their study by light microscopy. Mite identification was conducted based on original descriptions (Fonseca, 1936b, 1958; Savchenko and Lareschi, 2019, 2022).

### Molecular procedures

All mites were processed for DNA isolation. This procedure was applied individually to each mite using Chelex®100 following a non-destructive protocol (Savchenko and Lareschi, 2019). Two Polymerase Chain Reaction (PCR) were performed: one targeting the nuclear 18S-ITS1-5.8S-ITS2-28S region and another amplifying the mitochondrial cytochrome oxidase I (COI) gene. For the 18S-ITS1-5.8S-ITS2-28S region, PCR conditions consisted of an initial denaturation at 95 °C for 10 min, followed by 35 cycles of denaturation at 95 °C for 20 s, annealing at 51 °C for 30 s, extension at 72 °C for 90 s and a final extension at 72 °C for 10 min. The primers used were RhITS-F (5′-AGAGGAAGTAAAAGTCGTAACAAG-3′) and RhITS-R (5′-ATATGCTTAAATTCAGGGGG-3′) (De Rojas et al., 2002).

PCR amplification of COI followed a similar protocol, with an annealing temperature of 50 °C. The primers used were Kmt6 (5′-GGAGGATTTGGAAATTGATTAGTTCC-3′) and HC02198 (5′-TAAACTTCAGGGTGACCAAAAAATCA-3′) (Folmer et al., 1994; Zhu et al., 2015). PCR products were sequenced by Macrogen (Seoul, South Korea).

### Data analysis

Forward and reverse sequences were assembled, edited and aligned using CodonCode Aligner (CodonCode Corporation). The sequences generated for the 18S-ITS1-5.8S-ITS2-28S nuclear region and the COI gene were compared with sequences of laelapine mites available in the GenBank database, including 32 sequences of the nuclear region and 17 sequences of COI (see details in Table 1). Sequences of *Androlaelaps fahrenholzi* (Berlese, 1911), *Gigantolaelaps wolffsohni* (Oudemans, 1910), *Mysolaelaps microspinosus* Fonseca, 1936 and *Mysolaelaps parvispinosus* Fonseca, 1936 retrieved from GenBank were included to test relationships among laelapid genera. Sequences of *Dermanyssus gallinae* (De Geer, 1778) (Mesostigmata: Dermanyssidae) were used as outgroups.

Two separate alignments were constructed: one including 41 sequences of the 18S-ITS1-5.8S-ITS2-28S region and another including 41 sequences of the COI gene. These alignments were subsequently concatenated into a single dataset of approximately 1300 bp. Given the distinct evolutionary patterns of nuclear and mitochondrial markers, phylogenetic analyses were conducted using a partitioned dataset. Substitution models were selected independently for each dataset using jModelTest 2 (Darriba et al., 2012) under the Akaike information criterion (AIC) and the corrected AIC. The Hasegawa-Kishino-Yano (HKY) + I + G was selected for the 18S-ITS1-5.8S-ITS2-28S region, whereas General Time Reversible (GTR) + I + G was selected for COI.

Phylogenetic relationships were inferred from the concatenated dataset using both maximum likelihood (ML) and Bayesian inference (BI). ML analyses were performed in IQ-Tree (Minh et al., 2020) with 2000 bootstrap replicates, while BI analyses were conducted in MrBayes 3.2.7 (Ronquist et al., 2012) for 10 million generations, with the first 25% of trees discarded as burn-in. Phylogenetic trees were visualized using Figtree v1.4.4. The topology of the ML tree was selected and displayed the ML bootstrap values and Bayesian posterior probabilities.

Pairwise genetic distances among groups of mites, obtained from the phylogenetic tree, were calculated in MEGA11 using the alignments separately, and those results were expressed as percentages.

## Results

Of the 23 analysed mites, the following taxa were identified: 1 *A. fahrenholzi* from *C. callosus*, 3 *G. wolffsohni* (1 from *O. flavescens* and 2 from *O. nigripes*), 2 *L. echidninus* from *R. norvegicus*, 2 *Laelaps galliarii* Savchenko and Lareschi, 2022 from *O. nigripes*, 3 *L. manguinhosi* Fonseca, 1936 (2 from *H. brasiliensis* and 1 from *H. chacarius*), 1 *Laelaps mazzai* (Fonseca, 1939) from *C. sorella*, 1 *Laelaps odysseos* Savchenko and Lareschi, 2022 from *N. squamipes*, 2 *L. paulistanensis* Fonseca, 1936 from *O. nigripes*, 3 *Laelaps scapteromyos* Savchenko and Lareschi, 2022 from *S. aquaticus*, 1 *Laelaps schatzi* Lareschi and Savchenko, 2021 from *O. fornesi*, 2 *M. microspinosus* and 2 *M. parvispinosus*, all from *O. flavescens*. From these specimens, 8 sequences of the 18S-ITS1-5.8S-ITS2-28S nuclear region and 23 COI sequences were generated (Table 1).

Phylogenetic analyses of sequences generated in this study and those retrieved from GenBank (Table 1) revealed that all cricetid-associated mites formed a well-supported clade, separated from the murid-associated taxa. In contrast, the 3 species of murid-associated mites, *L. echidninus* (South America), *L. giganteus* and *L. muricola* (both from South Africa), did not form a well-supported monophyletic group, but instead were recovered as basal lineages separated from all the cricetid-associated *Laelaps*. Genetic distances between murid- and cricetid-associated laelapids ranged from 15% to 23% for the 18S-ITS1-5.8S-ITS2-28S region and from 19% to 25% for COI (Table 2).

**Table 2.** Genetic distances in percentage between clades of mites obtained from the phylogenetic tree for 18S-ITS1-5.8S-ITS2-28S and cytochrome oxidase I (COI) Table 2 long description.

	Mite clade (Host families and tribes)	1	2	3	4	5	6
1	*Laelaps* spp. (Cricetidae: tribes Oryzomyini and Akodontini)	—	7	10	20	16	20
2	*Mysolaelaps* spp. (Cricetidae: tribe Oryzomyini)	19	—	10	20	16	20
3	*Laelaps mazzai* (Cricetidae: tribe Phyllotini)	26	26	—	19	17	18
4	*Gigantolaelaps wolffsohni* (Cricetidae: tribe Oryzomyini)	23	24	25	—	19	23
5	*Androlaelaps fahrenholzi* (Cricetidae: tribe Phyllotini)	23	28	27	21	—	15
6	*Laelaps* spp. (Muridae: tribes Arvicanthini, Rattini, Praomyini)	20	22	25	19	19	—

Values above the diagonal indicate genetic distances of the 18S-ITS1-5.8S-ITS2-28S region and under the diagonal for COI.

**Figure 1.**
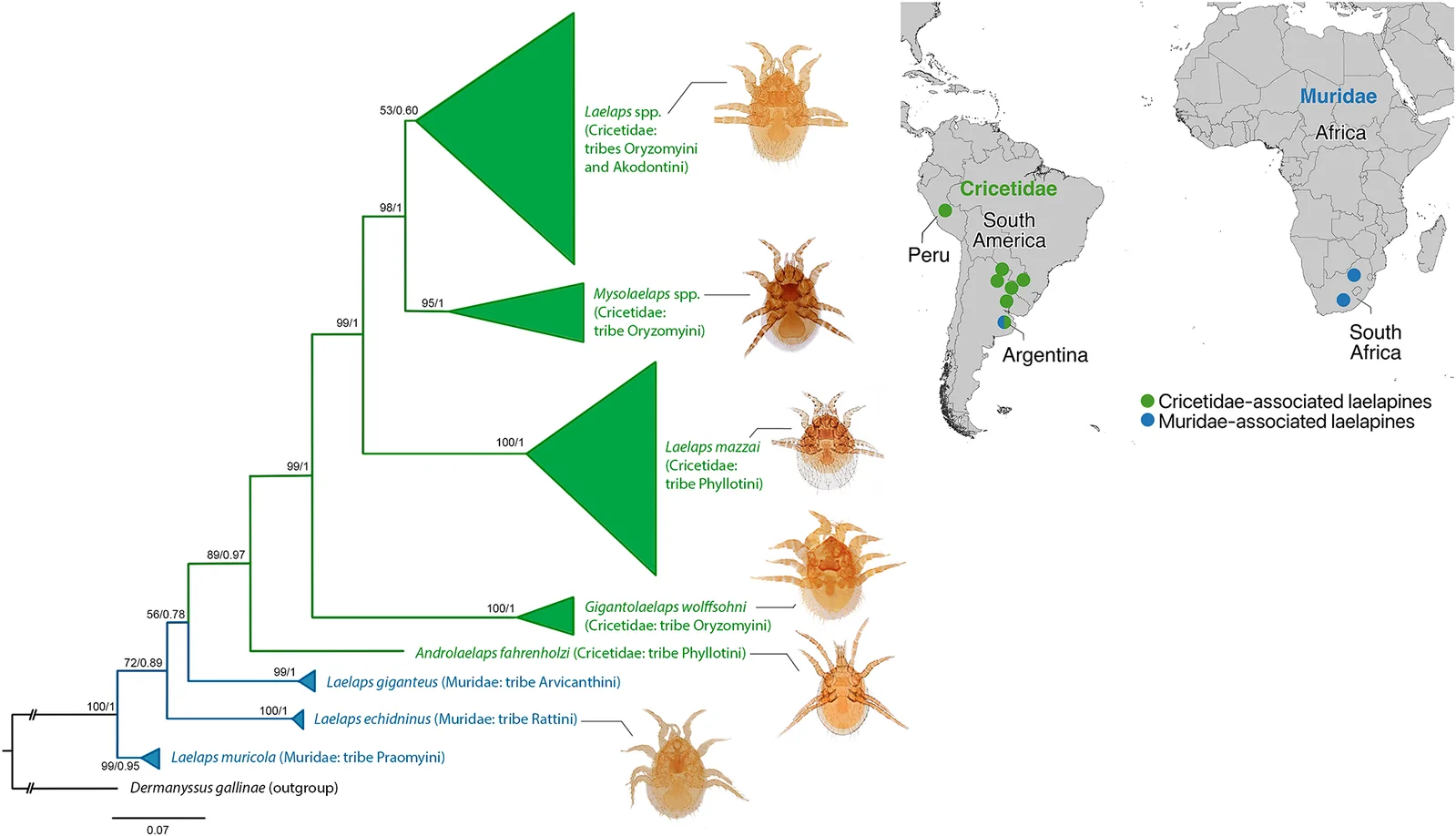
Phylogenetic tree based on concatenated nuclear (18S-ITS1-5.8S-ITS2-28S) and mitochondrial (COI) genes of laelapine mites from Muridae and Cricetidae rodents. The topology of the maximum likelihood (ML) tree was selected and displayed, on the nodes, the ML bootstrap values (left) and Bayesian posterior probabilities (BI, right). Branch colours and coloured circles on the map indicate host family associations. Coloured circles on the map represent approximate locations and are shown for reference only, not precise sampling points. Green denotes lineages associated with Cricetidae, whereas blue denotes lineages associated with Muridae. The presence of both colours in Argentina reflects the occurrence of cricetid-associated and murid-associated *Laelaps* lineages. Figure 1 long description.

Within the Cricetidae-associated clade, 5 subclades were recovered. One subclade included *A. fahrenholzi* associated with Phyllotini rodents and was recovered as sister of the remaining taxa. A second subclade comprised *G. wolffsohni* associated with Oryzomyini rodents. A third subclade included *L. mazzai*, also associated exclusively with Phyllotini. A fourth subclade grouped *Mysolaelaps* species associated with Oryzomyini rodents. The fifth subclade included *Laelaps* species associated with Oryzomyini and Akodontini rodents, namely *L. galliarii, L. paulistanensis, L. schatzi, L. manguinhosi, L. odysseos* and *L. scapteromyos*, though with weaker nodal support (Figure 1). Genetic distances between *Mysolaelaps* species and *Laelaps* associated with Oryzomyini rodents were lower (18S-ITS1-5.8S-ITS2-28S: 7%, COI: 19%) than distances between *L. mazzai* and *Laelaps* species associated with Oryzomyini and Akodontini rodents (18S-ITS1-5.8S-ITS2-28S: 10%, COI: 26%) (Table 2).

Overall, these results indicate that *Laelaps* is polyphyletic. Within Cricetidae, phylogenetic structure appears linked to host tribal affiliation, with distinct lineages recovered for Phyllotini, Akodontini and Oryzomyini hosts, respectively (Figure 1), separated by substantial genetic distances (18S-ITS1-5.8S-ITS2-28S: 18–20%, COI: 20–25%) (Table 2). The murid-associated lineages, while recovered outside the cricetid clade, lacked sufficient nodal support to be interpreted as a coherent monophyletic group.

## Discussion

The primary result of this study is the polyphyly of *Laelaps*, with cricetid-associated lineages forming a well-supported clade with a tendency for murid-associated taxa to be recovered separately. Within the cricetid-associated clade, phylogenetic structure appears linked to host tribal affiliation, with distinct lineages recovered for Phyllotini, Oryzomyini and Akodontini rodents. Notably, the entanglement of morphologically distinct genera (*Androlaelaps, Gigantolaelaps* and *Mysolaelaps*) within the cricetid-associated clade further demonstrates that current generic boundaries in Laelapidae do not reflect evolutionary history.

The murid-associated lineages, while recovered outside the cricetid clade, did not form a well-supported monophyletic group, and their phylogenetic relationships remain unresolved pending broader sampling. However, the substantial genetic distances between cricetid- and murid-associated laelapids are consistent with long-term independent diversification following the divergence of Cricetidae and Muridae around 25 Mya ago (e.g. Steppan and Schenk, 2017; López-antoñanzas et al., 2024). From a co-evolutionary perspective, such a temporal depth makes a long-term persistence of the same specialized parasite lineages across both families highly improbable (Hugot, 2007). The polyphyly of *Laelaps* revealed here is therefore consistent with this expectation and suggests that the traditional treatment of *Laelaps* as a single, widely distributed genus reflects historical reliance on morphologically conservative characters rather than genuine evolutionary continuity.

Since its description, *Laelaps* has been repeatedly revised, with successive authors refining its diagnostic limits while emphasizing morphological uniformity and ecological similarity across species (Zachvatkin, 1948; Zumpt, 1950; Bregetova, 1956). Although Tipton (1960) formalized the concept of *Laelaps sensu stricto*, other authors adopted a broader interpretation (*Laelaps sensu lato*) by retaining lineages separated by differences in chaetotaxy and shield morphology, including the treatment of *Echinolaelaps* as a subgenus of *Laelaps* (e.g. Strandtmann and Mitchell, 1963). The polyphyly recovered here is consistent with this long history of taxonomic instability and suggests that the morphological characters traditionally used to delimit *Laelaps* and related genera are evolutionarily labile and insufficient to capture phylogenetic structure. Notably, species such as *L. giganteus* and *L. muricola* were previously included within the *Echinolaelaps* complex together with *L. echidninus* (Strandtmann and Mitchell, 1963), yet our results show these murid-associated species occupy a very different phylogenetic position from the cricetid-associated lineages. Clarifying the molecular placement of additional species historically assigned to this complex will be essential to evaluate the potential resurrection of *Echinolaelaps* as a valid genus.

Within Cricetidae-associated mites, the paraphyly of *Laelaps* with respect to *Mysolaelaps* and *Gigantolaelaps* is also noteworthy. Although these genera are morphologically distinguishable based on major characters, particularly the shape and chaetotaxia of the genitoventral shield (see details in Fonseca, 1939, 1958), such differences appear insufficient to ensure taxonomic stability in the absence of molecular evidence. This situation illustrates a recurrent challenge in laelapid systematics: determining the taxonomic level at which morphological variation should be interpreted, whether at the species, genus or higher ranks (e.g. Furman, 1972b). Historically, these decisions have involved a considerable degree of subjectivity, contributing to the persistence of broadly defined and potentially artificial genera within Laelapidae (Dowling and OConnor, 2010). In *Laelaps*, this subjectivity is particularly evident in the interpretation of fine differences in idiosomal chaetotaxy and shield morphology, characters that have traditionally been considered sufficient to delimit genera despite their apparent evolutionary lability (Furman, 1971).

The case of *L. echidninus* associated with *R. norvegicus* in South America provides additional insights into parasite dynamics during host introductions. Despite the relatively recent introduction of *R. norvegicus* into the New World via human activities (Steppan et al., 2005), *L. echidninus* appears to have been retained by its host and shows no evidence of host switching to native sympatric cricetids. This pattern contrasts with expectations of the enemy release hypothesis, which predicts reduced parasite loads in introduced hosts (Torchin et al., 2003). Instead, our results are consistent with the view that highly host-specific parasites are more likely to persist during host range expansion, whereas generalist parasites are more prone to loss (Poulin, 1998; Schatz and Park, 2023). Similar patterns have been reported across diverse parasite groups, including arthropods, bacteria, protozoa and viruses, and have been described as uncommon but plausible outcomes of host introductions (Schatz and Park, 2023).

The absence of spillover to native rodents and the lack of spillback from cricetids to *Rattus* further emphasize the strong host specificity of *L. echidninus*, with only occasional accidental associations reported in the literature (Lareschi, 1996). In such cases, the presence of *L. echidninus* on the native rodent *S. aquaticus* was interpreted as the use of an alternative host in environments where this rodent is in sympatry with *Rattus* spp. in ecological disturbed environment (Lareschi, 1996). Indeed, *S. aquaticus* has been identified as a suitable alternative host for multiple laelapid mites in riparian environments of the Río de la Plata basin (Lareschi, 1996).

Within the cricetid-associated clade, *A. fahrenholzi* emerged as a sister lineage to the remaining taxa. This mite represents a complex species and has been suggested as a transitional stage between free-living predatory mites and obligate parasites (Radovsky, 1994; Dowling, 2006; Dowling and OConnor, 2010). As occurs with *Laelaps, Androlaelaps* is a genus that requires taxonomic revision. Additional sampling across host species and geographic regions will be necessary to clarify its evolutionary position.

The remaining mites formed a complex assemblage including *Gigantolaelaps, Mysolaelaps* and 2 lineages currently assigned to *Laelaps*, a pattern that directly illustrates the inadequacy of current generic boundaries. Within *Laelaps*, one lineage represented by *L. mazzai* was recovered exclusively in association with *Calomys* spp. (Phyllotini), whereas the other comprised species predominantly associated with Oryzomyini rodents (*L. galliarii, L. manguinhosi, L. odysseos, L. paulistanensis* and *L. schatzi*), with the exception of *L. scapteromyos* associated with *S. aquaticus* (Akodontini). The fact that *Gigantolaelaps* and *Mysolaelaps*, morphologically distinct genera (Fonseca, 1939, 1958; Furman, 1972b), are recovered as more closely related to some *Laelaps* lineages than those *Laelaps* lineages are to each other further underscores that morphological characters alone are insufficient to delimit natural groups within Laelapidae. Some of these lineages do exhibit marked morphological differences (Savchenko and Lareschi, 2022), but others, such as *L. mazzai*, remain difficult to distinguish using traditional characters, likely explaining their long-standing inclusion within a broadly defined *Laelaps*.

The phylogenetic distinctiveness of *L. mazzai* is particularly noteworthy given its taxonomic history. This species was previously proposed as belonging to a separate genus, *Schistolaelaps* Fonseca, 1959, based on morphological differences including a hypertrichous idiosoma, but was later re-included within *Laelaps* when those differences were considered insufficient at the generic level (Furman, 1971, 1972b). Interestingly, Dowling and OConnor (2010) had already flagged this taxon as warranting separate generic recognition based on their broader phylogenetic analysis of Dermanyssoidea. Our molecular results, with strong nodal support for the *L. mazzai* lineage, corroborate this interpretation and lend support to the revalidation of *Schistolaelaps* for species of *Laelaps* associated with Phyllotini rodents of the genus *Calomys*.

The polyphyly of *Laelaps* recovered here underscores the need for a comprehensive taxonomic revision of Laelapidae integrating molecular, morphological and ecological data. Based on the evidence available, at least 3 distinct evolutionary entities can be recognized within what is currently treated as *Laelaps*: lineages associated with Muridae (though their monophyly remains unresolved), lineages associated with Cricetidae–Oryzomyini and Akodontini, and lineages associated with Phyllotini (*Calomys*), the latter likely corresponding to the revalidated genus *Schistolaelaps*. More broadly, these findings challenge the validity of other parasite taxa-associated murid and cricetid rodents that have traditionally been regarded as cosmopolitan based solely on morphological similarity, such as *A. fahrenholzi* and *Ornithonyssus bacoti* (Hirst, 1913). Both remain poorly explored from a phylogenetic perspective and, in light of our results, clearly warrant further integrative study (Till, 1963; Radovsky, 1994; Dowling and OConnor, 2010).

From an epidemiological perspective, the persistence of *L. echidninus* on introduced *Rattus* populations is noteworthy. This mite has been associated with bacteria of the genera *Anaplasma* and *Rickettsia* in Asia and Europe (Mit’ková et al., 2015; Kuo et al., 2020). Given the wide distribution of *R. norvegicus*, further studies are needed to assess the presence and potential public health relevance of these bacteria in South America.

In conclusion, molecular phylogenetic analyses demonstrate that *Laelaps* is polyphyletic, with a well-supported clade of cricetid-associated lineages clearly separated from murid-associated taxa. Within Cricetidae, phylogenetic structure is linked to host tribal affiliation, and the entanglement of morphologically distinct genera, *Androlaelaps, Gigantolaelaps* and *Mysolaelaps*, within the cricetid clade demonstrates that current generic boundaries in Laelapidae do not reflect evolutionary history. These results support the polyphyly of *Laelaps* and consequently challenge the wide distribution of this mite, highlighting the need for an integrative taxonomic revision of the species, combining molecular, morphological and ecological data.

## Supporting information

10.1017/S0031182026102224.sm001 Espinoza-Carniglia et al. supplementary material Espinoza-Carniglia et al. supplementary material

## Data Availability

The original data generated in this study are available in GenBank (National Center for Biotechnology Information, https://www.ncbi.nlm.nih.gov/genbank/) under the following accession numbers: OQ241929, OQ241930, OQ241937, OQ241939–OQ241943, OR405892–OR405895, OR405898, PV952202, PV952205–PV952221.

## Supplementary material

The supplementary material for this article can be found at https://doi.org/10.1017/S0031182026102224.

## Table 1 Long description

The table lists mite taxa used for phylogenetic sampling, paired with their host species, host family and tribe, country of mite origin, and accession numbers for a nuclear ribosomal region and the COI gene. Sampling is dominated by Laelaps species associated with rodents, especially Cricetidae and Muridae, with most records originating from Argentina and several from South Africa, Italy, Israel, and Peru. Dermanyssus gallinae on domestic chicken is included as an outgroup, with one Italian record providing a nuclear accession and one Israeli record providing a COI accession, indicating missing data across markers for that species. Several Laelaps species have multiple entries reflecting different hosts or multiple sequences per marker, such as Laelaps mazzai across several Calomys hosts in Argentina plus one Peruvian record. Many COI accessions are newly generated in the study, while some nuclear and COI sequences are drawn from prior publications. Missing values are explicitly indicated for some marker-by-record combinations, so comparisons across taxa may rely on different gene coverage.

Navigate back to Table 1.

## Table 2 Long description

The table reports pairwise genetic distances, in percent, among six mite clades linked to different rodent host families and tribes. For each clade pair, the upper-right half gives distances for the 18S-ITS1-5.8S-ITS2-28S region, and the lower-left half gives distances for COI. In the 18S-ITS region, the smallest distance is 7 percent between clades 1 and 2, and most other values fall between 10 and 23 percent, with several pairs at 20 percent. In COI, distances are generally higher, ranging from 19 to 28 percent, with the largest distance between clades 2 and 5 at 28 percent. COI also shows 26 percent between clade 3 and clades 1 and 2, and 25 percent between clade 6 and clades 3 and 4. The lowest COI distance is 19 percent, occurring for multiple pairs including clades 1 and 2, clades 1 and 6, clades 4 and 6, and clades 5 and 6. Overall, COI indicates greater divergence among clades than the 18S-ITS region, but comparisons must use the correct half of the matrix because each marker is reported in a different triangle.

Navigate back to Table 2.

## Figure 1 Long description

The diagram is a phylogenetic tree with a map indicating host associations of laelapine mites with Cricetidae and Muridae rodents. The tree is oriented vertically, with branches extending from the bottom to the top. The left side of the tree features green branches representing lineages associated with Cricetidae, while blue branches on the right represent lineages associated with Muridae. Each branch is labeled with bootstrap values and Bayesian posterior probabilities. The topmost green clade includes Laelaps species associated with Oryzomyini and Akodontini rodents. Below it, another green clade represents Mysolaelaps species associated with Oryzomyini. Further down, a green clade includes Laelaps mazzai associated with Phyllotini. The lowest green clade groups G. wolffsohni with Oryzomyini. The blue clade at the bottom includes Laelaps species associated with Muridae, such as L. giganteus and L. muricola. The map on the right shows approximate locations of these associations in South America and South Africa, with green circles for Cricetidae and blue circles for Muridae. The presence of both colors in Argentina indicates the occurrence of both cricetid-associated and murid-associated lineages.

Navigate back to Figure 1.
