# Supplementary material for: Molecular evidence of rodent-associated mites tests the monophyly and challenges the cosmopolitan status of Laelaps Koch, 1836 (Mesostigmata: Laelapidae)
Source: Parasitology. 2026 Jun 8;153(7):930–7. doi: 10.1017/S0031182026102224 (PMC13735554; doi:10.1017/S0031182026102224)
Supplement: Supplementary file 1 [file S0031182026102224sup001.pdf]

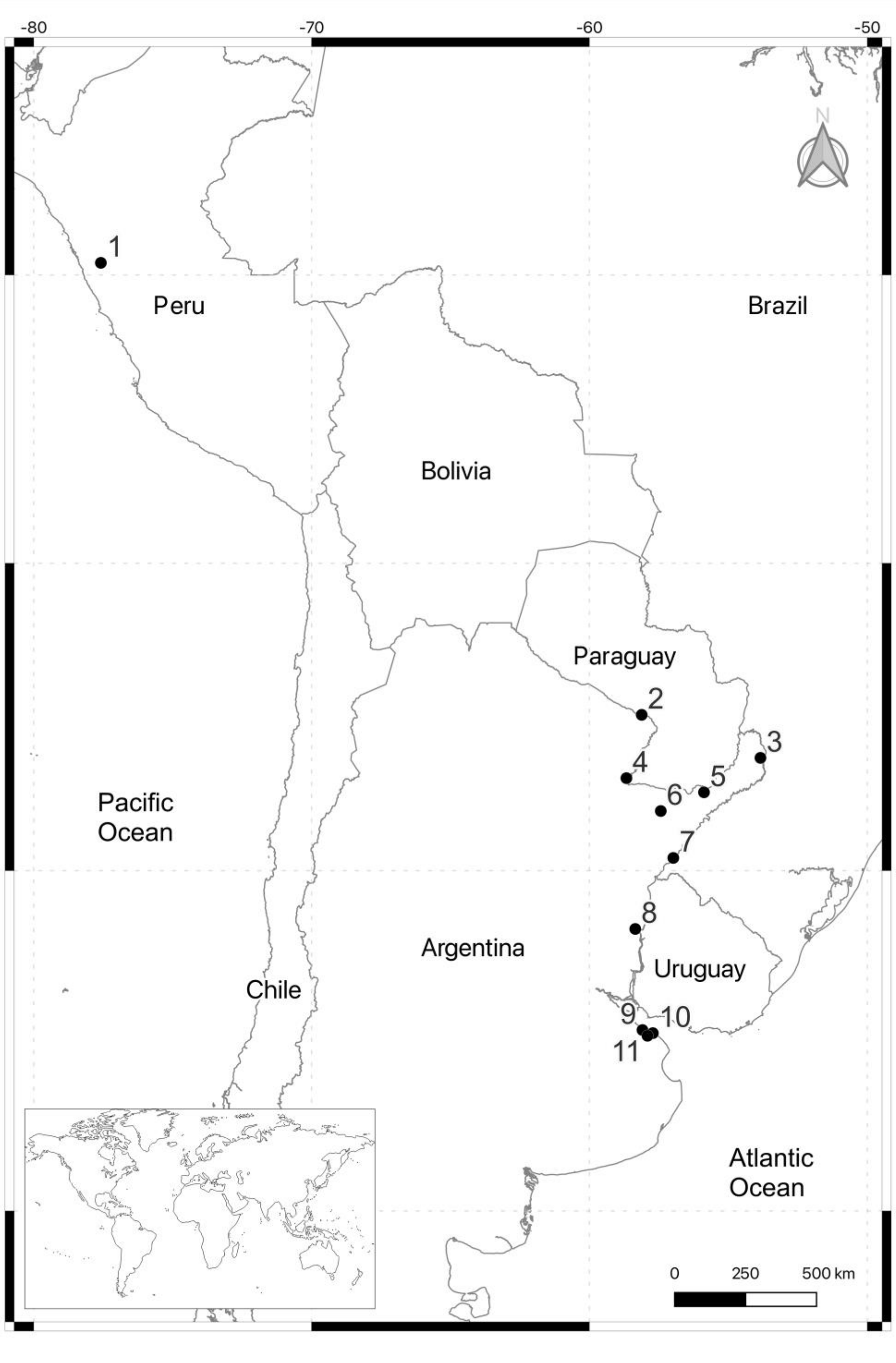

Figure S1. Localities of laelapine mites used in this study from Muridae and Cricetidae rodents. Localities: 1) Comunidad 24 de Junio, Peru; 2) INTA IPAF-NEA, Ruta Provincial Nº8, Paraje Isla Puen S/N, Laguna Naineck, Departamento Pilcomayo; 3) Parque Provincial Piñalito, Departamento San Pedro;

4) 5 km NW de Puerto Las Palmas, Departamento Bermejo; 5) Estancia Santa Inés, RP 105, Km 8.5, Departamento Capital; 6) Portal San Nicolás (ex Estancia San Nicolás), Parque Nacional Iberá, 27 km al SE de San Miguel, Departamento San Miguel; 7) Reserva Santo Domingo 20 km al N de Paso de los Libres; 8) Ruta Nacional 130, km 9, Villa Elisa, Departamento Colón; 9) Pereyra, Partido de Berazategui; 10) La Balandra, Partido de Berisso; 11) Calle 143 y Diagonal 630, Arana, Partido de La Plata.
